# Experimental vibration dataset collected of a beam reinforced with masses under different health conditions

**DOI:** 10.1016/j.dib.2024.110043

**Published:** 2024-01-11

**Authors:** Amanda A.S.R. de Sousa, Marcela R. Machado

**Affiliations:** a Department of Mechanical Engineering, University of Brasilia, 70910-900, Brasília, Brazil; b Faculty of Civil, Environmental Engineering and Architecture, Bydgoszcz University of Science and Technology, Sylwestra Kaliskiego 7, Bydgoszcz, Poland

**Keywords:** Structural health monitoring, Damage detection, Experimental practices, Uncertainty quantification, Machine learning

## Abstract

Vibration signals extracted from structures across diverse health conditions have become indispensable for monitoring structural integrity. These datasets represent a resource for real-time condition monitoring, enabling the precise detection and diagnosis of system anomalies. This paper aims to enrich the scientific community's database on structural dynamics and experimental methodologies pertinent to system modelling. Leveraging experimental measurements obtained from mass-reinforced beams, these datasets validate numerical models, refine identification techniques, quantify uncertainties, and continuously foster machine learning algorithms' evolution to monitor structural integrity. Furthermore, the beam dataset is data-driven and can be used to develop and test innovative structural health monitoring strategies, specifically identifying damages and anomalies within intricate structural frameworks. Supplemental datasets like Mass-position and damage index introduce parametric uncertainty into experimental and damage identification metrics. Thereby offering valuable insights to elevate the efficacy of monitoring and control techniques. These comprehensive tests also encapsulate paramedic uncertainty, providing robust support for applications in uncertainty quantification, stochastic modelling, and supervised and unsupervised machine learning methodologies.

Specifications Table

**Table:** 

Subject	Mechanical / Civil Engineering
Specific subject area	Dynamic, vibration, uncertainty quantification, machine learning, and structural monitoring
Data format	Raw and Analysed
Type of data	Table, MATLAB code, dataset. .txt file (dataset with vibration test) .xlsx file (dataset with numbers containing DIs and random position), .mat (code to read the measurement and random sampling)
Data collection	There are three datasets named Beam-signal, Mass-position, and DIs. The dataset beam-signal consists of the beam's inertance response magnitude and phase. Excitation of the structure was conducted by a Modal impact hammer (PCB 086CO3). The structure dynamic response was measured with an accelerometer (PCB 353B03). The setup includes a Polytec acquisition system, Polytec VibSoft-20, analogue 2-channel with frequencies from DC up to 20 kHz. The dataset beam-signal contains multiple inertance measurements acquired with an accelerometer mounted at the free end of the beam reinforced with masses. The impact excitation was introduced close to the clamped edge. In each measurement, the attached mass was placed in the position randomly generated to add some variability to the system. For the four integrity conditions of the structure (Health, damaged-2.95, damaged-5.92, and damaged-8.84), the reference inertance (acceleration) response assumed the deterministic mass positions. The dataset Mass-position consists of a random sampling position of the six masses attached to the beam. Seventy samples of each mass position were generated in a Matlab code considering Uniform distribution with zero mean and unitary standard deviation. Dataset DIs contain 280 samples of damage index calculated with FRAC by considering the reference inertance response as the deterministic mass position of the structure in a health state condition against the inertance measured considering the random mass position with health and damaged conditions. All three data available are formats raw magnitude and phase data measurement (*.txt), computed damage indexes (*.xlsx, and *.mat), and simulated random samples(*.xlsx, and *.mat).
Data source location	Data was collected at the Vibration Laboratory of the Department of Mechanical Engineering at the University of Brasília, Brasília, Distrito Federal, Brazil.
Data accessibility	Repository name: Zenodo [2]. Data identification number: https://doi.org/10.5281/zenodo.8081690 Direct URL to data: https://zenodo.org/records/8081690
Related research article	A. A. S. R. de Sousa, J. d. S. Coelho, M. R. Machado, M. Dutkiewicz, Multiclass supervised machine learning algorithms applied to damage and assessment using beam dynamic response, Journal of Vibration Engineering Technologies (2023). doi: https://doi.org/10.1007/s42417-023-01072-7

## 1 Value of the Data


 

• The dataset beam-signal contains the magnitude and phase of the spectrum vibration signals in the frequency domain measured from a beam reinforced with masses under healthy and faulty conditions. This data is for a commonly used system in various industrial applications. The data can be used for online condition process monitoring to detect and diagnose any anomaly or faulty condition in the system.
• The datasets are intended to benefit the scientific community investigating the dynamics of structures and readers interested in experimental practices applied to systems and modelling. These datasets can be employed for numerical model validation, identification techniques, uncertainty quantification, machine learning, and structural integrity monitoring algorithms based on experimental measurements.
• Dataset beam can be used to develop structural health monitoring techniques for detecting damage and anomalies in the structure. The datasets Mass-position and DIs used to impose the parametric uncertainty in the experimental and damage identification metrics can be used for further insights on new monitoring and control techniques, respectively. Since the tests include paramedic uncertainty, they can also be employed in uncertainty quantification, stochastic modelling, and supervised and unsupervised machine learning techniques.

## 2 Background

In structural health monitoring, a common approach involves monitoring, locating, categorising, estimating the severity and prognosis of structural integrity. However, establishing techniques that comprehensively cover all these aspects simultaneously within a single monitoring algorithm, exhibiting strong generalisation abilities for multi-task monitoring using vibration-based signals, remains a significant challenge. Additionally, the inherent variabilities and uncertainties within the system further compound the complexity of the structural health monitoring process [5,6]. To overcome certain monitoring processes, the present dataset has been acquired to support and evaluate the methodologies proposed in [1,3,4] and structural health monitoring techniques based on vibration signature. Methodologies that integrate vibration physics-based models and data-driven involve preprocessing and feature selection of the dataset inputs to facilitate the training and validation that will benefit from these datasets. Furthermore, machine learning techniques, uncertainty quantification and stochastic approaches for pattern recognition and structural health monitoring can employ this dataset for validation, testing, and modelling purposes.

## 3 Data Description

This article provides an explanatory description of the experiments that collect vibration signals of a beam reinforced with masses under different health conditions and three levels of damage, which is considered the beam's mass loss. The vibration signals are collected from the accelerometer attached to the driven point mounted at the beam free-end, as shown in Fig. 5. The collected data includes 280 inertance responses' magnitudes and respective phases considering health and damage conditions, 70 sampling positions for the six masses attached to the beam, and 280 damage indices calculated with the FRAC method. The data collection is organised in three datasets named Beam-signal, Mass-position, and DIs.

### 3.1 Dataset mass-position

**Fig. 1:**
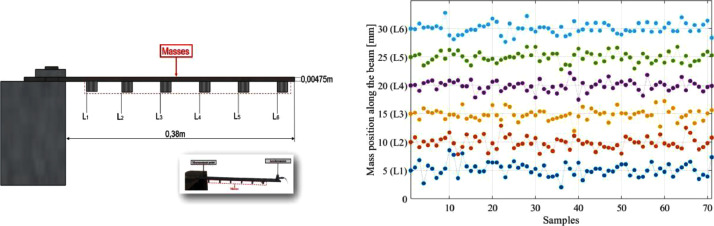
Graphic representation of the mass position of the magnets attached to the beam (LHS). Random masses location samples for each attached mass (RHS).

In each measurement, the six masses are positioned at different places along the beam, as shown in Fig. 1. The masses' position is considered a random variable under the support of Uniform distribution. The mean value is the deterministic value of the masses' positions, and the coefficient of variation is assumed to be 10 %. The dataset comprises seventy random positions of each mass organised in an Excel file (Mass position.xlsx), illustrated in Fig. 1(RHS). The deterministic values of the position L1 to L6 are considered the mean value for the randomly sampled generation. Thus, the masses 1, 2, 3, 4, 5, and 6 are located at the deterministic positions of L1 = 5 cm, L2 = 10 cm, L3 = 15 cm, L4 = 20 cm, L5 = 25 cm, L6 = 30 cm, respectively. This paper also provides the Matlab routine in Appendix 1 for the position data generation. Therefore, as in each measurement, some variability is included in the experiment by repositioning the masses' positions.

### 3.2 Dataset beam-signal

The dataset named Beam-signal comprises experiments that collected vibration signals from a beam reinforced with masses. This dataset is provided in Text format (*.txt) and contains frequency vibration acceleration Magnitude (dB) and Phase (degrees) signals, along with the frequency range (Hz). The spectrum was measured over a frequency range of 0 to 2000 Hz with 6400 equally spaced frequency points (the number of points is 6400, and frequency discretisation is 0.3125) to capture the beam's closely spaced frequencies and obtain well-defined resonate peaks. The data was acquired under four different operating conditions:' Healthy', where all six masses are attached;' Damaged-2.96,' where the mass at position L1 is removed;' Damaged-5.92,' without masses at positions L1 and L3; and' Damaged-8.84,' without masses at positions L1, L3, and L5. Each experiment set was repeated seventy times for each structural condition, resulting in 240 samples when each measurement assumed the respective random mass position.

**Fig. 2:**
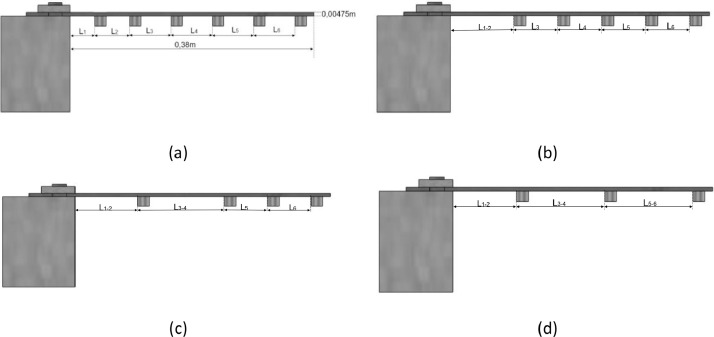
Graphic representation of the tests carried out in the experiment. (a) Undamaged beam with reinforced masses; (b) Damaged beam with mass loss of 2.96 % of the total mass; (c) Damaged beam with mass loss of 5,92 % of the total mass; (d)Damaged beam with mass loss of 8,84 % of the total mass.

Fig. 2 displays the structure in its healthy state and the three damaged conditions, considering damage as the mass loss of the reinforcements. Fig. 2a illustrates the experimental setup when the beam is in a healthy condition, with six masses positioned at distances of 5 cm from the clamped edge. Fig. 2b depicts the first damaged beam condition, demonstrating a mass loss of 2.96 % of the total mass, achieved by removing the mass at position L1. Subsequently, Fig. 2c represents a second damaged beam condition with a mass loss of 5.92 % by removing masses from positions L1 and L3. Lastly, Fig. 2d illustrates the third damaged beam condition, exhibiting a mass loss of 8.84 % by removing masses from positions L1, L3, and L5.

**Fig. 3:**
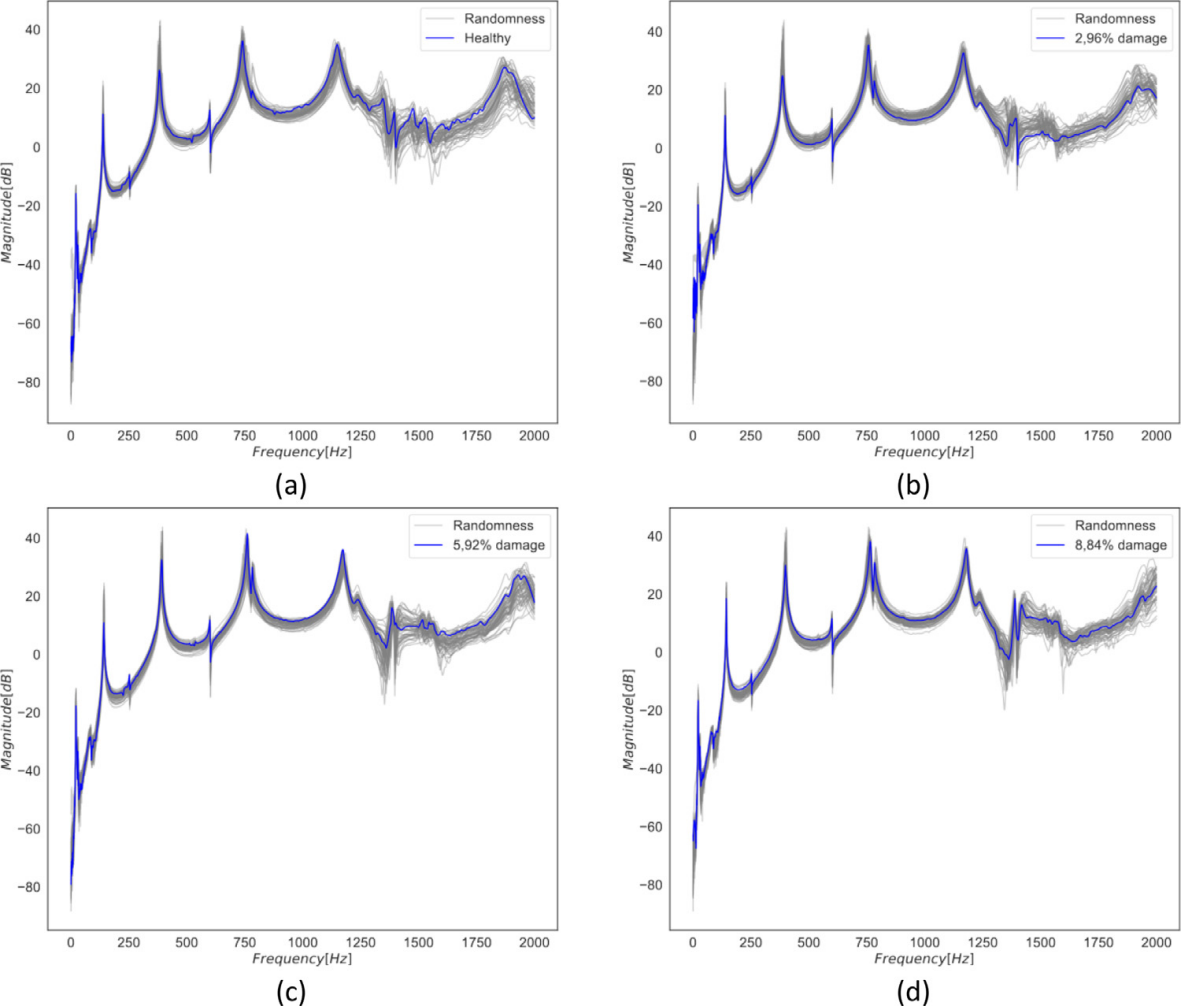
Experimentally measured inertance FRFs of the beam with reinforced mass. The blue curve is the deterministic position of the masses, and the grey curve considers randomness in the mass position. (a) Undamaged beam FRFs; (b) Damaged beam FRFs with a mass loss of 2.96 % of total mass; (c) Damaged beam FRFs with mass loss of 5.92 % of total mass; and (d) Damaged beam FRFs with a mass loss of 8.84 % of total mass.

The reduction in beam rigidity and mass due to the loss of reinforcement induces changes in resonance frequencies, consequently affecting the dynamic response. Fig. 3 illustrates the effect of gradual mass loss on the inertance frequency response function (FRF) of the beam considering mass randomly positioned, where the blue curve is the deterministic position of the masses, and the grey curve is seventy measurements considering randomness in the mass position. Fig. 3a shows the inertance response of the undamaged beams, while Fig. 3b represents the damaged beam with a mass loss of 2.96 % of the total mass. Fig. 3c shows the damaged beam with a mass loss of 5.92 %, and Fig. 3d exhibits the damaged beam with a mass loss of 8.84 %.

The Beam-signal dataset, available in [2], is accompanied by four folders and its name is given as 'Dataset Beam-signal Healthy', 'Dataset Beam-signal Damaged−2.96′, 'Dataset Beam-signal Damaged−5.92′, and 'Dataset Beam-signal Damaged−8.84′, respectively. The data containing the inertance magnitude and phase signal are collected in folders named as follows:

• **P-X**: The vibration signal of the beam under healthy conditions; here, '**X**' varies from 1 to 70, meaning the random samples measured assume different mass positions. P-1 considered the deterministic position of the masses.
• **P-X-1**: The vibration signal of the damaged beam with 2.96 % mass loss, '**X**' varies from 1 to 70, meaning the random samples measured assume different mass positions. **P-X-1** considered the deterministic position of the masses.
• **P-X-2**: The vibration signal of the damaged beam with 5.92 % mass loss, '**X**' varies from 1 to 70, meaning the random samples measured assume different mass positions. **P-X-2** considered the deterministic position of the masses.
• **P-X-3**: The vibration signal of the damaged beam with 8.84 % mass loss, '**X**' varies from 1 to 70, meaning the random samples measured assume different mass positions. **P-X-3** considered the deterministic position of the masses.

### 3.3 Dataset DI

Structural damage influences the dynamic response of physical beams, consequently, it can be employed to calculate damage indices (DI). The inertance response presented in Section 1.2 is then employed to calculate the damage indices. The Frequency Response Assurance Criterion (FRAC) is used in this work. FRAC is a damage index that correlates FRF signals, where a strong correlation is indicated by a unity representing no damage state. In contrast, the lowest correlation to zero means damage condition and severity. Eq. (1) formulates the FRAC that compares the FRF signal of the damaged beam ($H_{ij}^{d}$) and the healthy beam indicated by ($H_{ij}^{u}$). The DI can detect and quantify the damage because the damage directly influences the system's vibration.

(1) $$FRAC_{ij}\left(\omega \right)=\frac{{\left[H_{ij}^{d}\left(\omega \right){\left(H_{ij}^{u}\left(\omega \right)\right)}^{*}\right]}^{2}}{\left[H_{ij}^{u}\left(\omega \right){\left(H_{ij}^{u}\left(\omega \right)\right)}^{*}\right]\left[H_{ij}^{d}\left(\omega \right){\left(H_{ij}^{d}\left(\omega \right)\right)}^{*}\right]}$$

where ∗ defines the complex conjugate operator. The excitation is applied at the *j*th coordinate, and the response function is at the *i*th coordinate. The index compares the FRFs of damaged and healthy beams, the entire spectrum of energy response information.

The change in dynamic characteristics of the beam reinforced with masses can be used as damage indicators. The FRAC performs a better damage indicator in a numerical study because it uses the spectrum energy to the whole response signal. Hence, FRAC calculates the DIs using the experimental dataset. The DIs are estimated by correlating the inertance responses of undamaged and damaged beam conditions with the removal of 2.96, 5.92, and 8.84 % of the total mass of the beam. The inertance responses of the undamaged with deterministic masses positions are considered the reference spectrum signal correlated to the responses of undamaged and damaged with a random mass location.

**Fig. 4:**
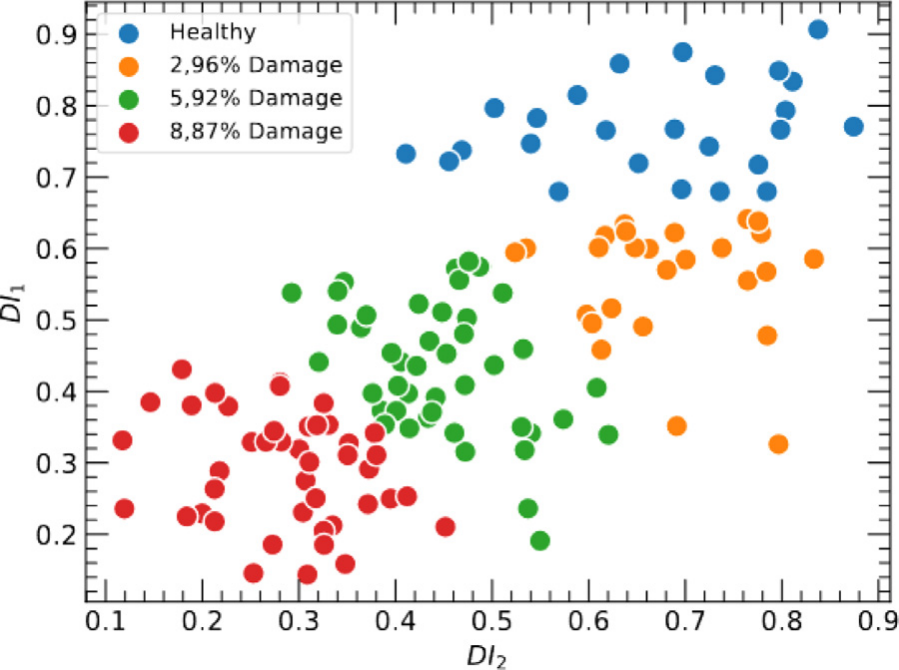
Scatter plot correlating FRAC DIs groups samples dataset obtained for the beam with reinforced masses for different mass loss.

Monitoring damage using vibrational-based responses within this damage level is critical because the perturbation is minimal in the mode shapes. The dataset DIs can be clustered by associating the data balancing. Fig. 4 shows the scatter plot of the provided dataset and the correlation between FRAC DI1 and FRAC DI2, where DI1 and DI2 are two columns of DIs used to correlate the data. The DI values cluster around 0.1 and 0.9, demonstrating that the DI data values have a high correlation and can indicate strong correction between the data. This result demonstrated the DI estimation and an application of the provided data set that can be employed in supervised and unsupervised machine learning algorithms and damage pattern recognition.

Dataset DI named DI FRAC Exp-estimation file (*.xlsx) in [2] contains 280 Damage indices estimated with FRAC DI and clustered in two columns named DI-1 and DI-2. The damage is assumed to be a mass loss, which was set as 0 for the healthy condition, 2.96, 5.92, and 8.87 % for the damaged condition. Those values are respective to the total structure mass loss. The column "Multiclass classification" is the assumed label for machine learning applications.

## 4 Experimental Design, Materials and Methods

**Fig. 5:**
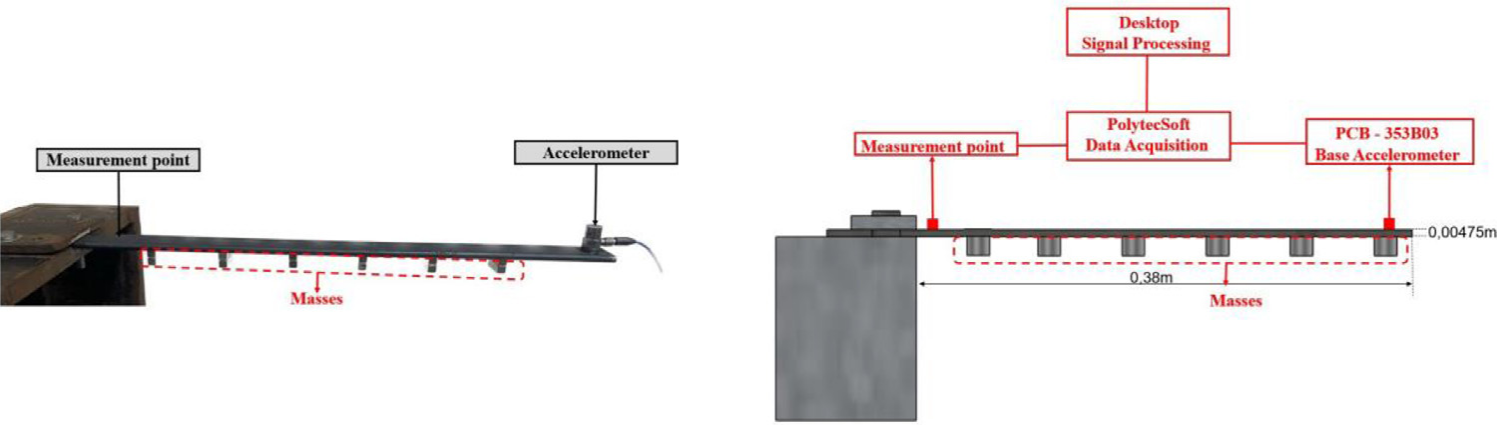
Physical beam attached with masses (LHS) and schematic experiment representation (RHS).

The physical structure consists of a cantilever beam with six attached masses along its length used to reinforce the beam. The experimental setup, shown in Fig. 5, consists of a steel cantilever beam of length L = 0.38 m, a width of 0.0254 m, and a height of 0.00475 m. Material properties are Young's modulus of 2.1 GPa and density of 7800 kg/m3. The reinforcement masses in a total of six neodymium magnets comprise 10.41 % of the total mass of the reinforced beam, which weighs 429.37 g. The beam is excited near the clamped edge with an impact hammer PCB 086CO3, and the acceleration response is acquired at the free edge of the beam by an accelerometer PCB 353B03. According to the manufacturer's specification the accelerometer Sensitivity is (±5 %)10 mV/g (1.02 mV/(m/s²)), measurement range of ±500 g pk (±4905 m/s² pk), broadband resolution of 0.003 g rms (0.03 m/s² rms), frequency range is (±5 %)1 to 7000 Hz, the sensing element is Quartz, and weight 0.38 oz (10.5 gm). The acquisition system is PolytecSoft, which provides the inertance response amplitude and phase.

Fig. 5 is considered the undamaged state of the beam reinforced with masses. The damage condition of the structure is considered by losing the mass of the reinforcement. Two hundred-eight measurements were performed in the beam, considering health and damage conditions. The six masses are positioned at different places along the beam in each measurement, as described in Section 1.1. The masses' position is considered a random variable under the support of Uniform distribution. The mean value is the deterministic value of the masses' positions shown in Fig. 2a, and the coefficient of variation is assumed to be 10 %.

## Limitations

Not applicable

## Ethics Statement

The authors have read and follow the ethical requirements for publication in Data in Brief and confirming that the current work does not involve human subjects, animal experiments, or any data collected from social media platforms.

## CRediT authorship contribution statement

**Amanda A.S.R. de Sousa:** Conceptualization, Formal analysis, Software, Validation, Visualization, Writing – original draft. **Marcela R. Machado:** Funding acquisition, Investigation, Methodology, Project administration, Resources, Software, Supervision, Writing – review & editing.

## Figures and Tables

**Table:** 

**Table:** 

## Data Availability

Damage assessment of a physical beam reinforced with masses - dataset (Original data) (Zenodo)

## APPENDIX

### Appendix A1. Routine for generation of the magnets' random position

This appendix contains the Matlab routine for mass position data generation. The mean value of the mass position is described in line 1 of the code, and the random generation is in line 3. This routine also plots the random realisations of the six mass positions at line 6.

Code 1: MATLAB Code for Generating Random Masses Positions

### Appendix A2. Routine to generate DI FRAC

In the present appendix, a Matlab routine for processing the dataset Beam-signal is provided. Lines 3 to 11, provide the function to load the data and truncate the data for healthy and damaged beams. The damage index FRAC is evaluated from lines 16 to 24, where a value between zero and unity is returned. In this experimental case, the structural damage influences the dynamic response of physical beams in higher frequency ranges. Consequently, for calculating Damage Indices (DI), a frequency band comprising the second, third, and fourth mode shapes most impacted by the structural damage. Therefore, a signal truncation can be applied, and it is included in the code.

Code 2: MATLAB Code for FRAC damage index calculation
